# Supplementary material for: Content-rich biological network constructed by mining PubMed abstracts
Source: BMC Bioinformatics. 2004 Oct 8;5:147. doi: 10.1186/1471-2105-5-147 (PMC528731; doi:10.1186/1471-2105-5-147)
Supplement: Additional File 5 — The original Chilibot query results of the term "long-term potentiation (LTP)" and 22 other terms, limiting the latest references analyzed to the years 1990, 1995, 2000, and 2004. [file 1471-2105-5-147-S5.bz2 › chilibotAdditionalFile5/ltp2000/html/PLC_ERK.html]

 


 **PLC** and **ERK** 
  
Found 34 abstracts in PubMed,  **30 abstracts were retrieved and analyzed**.  


---

 Search Google  |
 PDF files only 
|  EDU domain only 

---

**Interactive relationship** (e.g. stimulation, inhibition, etc)

- The inhibition of the  **ERK**  response was also observed in phospholipase C gamma2 deficient DT40 B cells, and expression of RasN17 in the  **PLC**  gamma2 deficient cells completely abrogated the  **ERK**  activation.  Ref: 9763608 J Exp Med, 1998
- However, neither inhibition of PI  **PLC**  nor chelation of calcium significantly reduced ATP stimulated  **ERK**  activity.  Ref: 10341225 J Neurosci, 1999
- Moreover, deficiency in fyn dependent  **PLC**  gamma1 catalytic activity may contribute to reduced PKC alpha dependent  **ERK**  activation.  Ref: 11093170 Eur J Immunol, 2000
- The treatment with phosphatidylcholine specific phospholipase C PC  **PLC**  inhibited macrophage proliferation, induced the expression of cytokines, and triggered a pattern of  **ERK**  activation equivalent to that induced by LPS.  Ref: 10702314 J Biol Chem, 2000
- These results indicate that G i o protein, tyrosine kinase, P13K, PKC, and  **PLC**  calcium... act upstream of fMLP stimulated  **ERK**  activation.  Ref: 10413116 FEBS Lett, 1999
- In addition, both the phospholipase C  **PLC**  inhibitor U73122 and the calcium chelator BAPTA attenuated  **ERK**  activation.  Ref: 10413116 FEBS Lett, 1999
- Because occupancy of P2Y receptors also leads to inositol phosphate formation, calcium mobilization, and PKC activation, we explored the possibility that signaling from P2Y receptors to  **ERK**  is mediated by a phosphatidylinositol specific phospholipase C PI  **PLC**  calcium pathway.  Ref: 10341225 J Neurosci, 1999
- Consistently, the transactivating potential of the C terminal domain of Elk 1, which is activated upon  **ERK**  mediated phosphorylation, was strongly induced in serum starved cells expressing v Ras or PC  **PLC** .  Ref: 9111071 J Biol Chem, 1997
- LPS induced  **ERK**  kinase activation was inhibited by tricyclodecan 9 yl xanthogenate D609, a relatively specific inhibitor of PC  **PLC** .  Ref: 10072552 J Immunol, 1999
- Similarly, the regulation of  **ERK**  and JNK responses involves both  **PLC**  gamma 2 and GTPases pathways.  Ref: 10358768 Annu Rev Immunol, 1999
- Hypoosmotic  **Erk**  phosphorylation was blocked by the MEK inhibitor PD098059 but was resistant to depletion of extracellular calcium and to inhibition of  **PLC** , PKC, erbstatin sensitive tyrosine kinases and elevation of intracellular cAMP.  Ref: 9705150 Biol Chem, 1998

**Parallel relationship** (e.g. studied together, co-existance, homology, etc.)

- We report here that extracellular signal regulated kinase  **ERK**  1 and 2 are constitutively activated in v Ras and PC  **PLC**  transformed cells in the absence of added growth factors.  Ref: 9111071 J Biol Chem, 1997
- The  **PLC**  gamma2 dependency of  **ERK**  activation was most likely due to protein kinase C activation rather than calcium mobilization, since loss of inositol, 5 trisphosphate receptors did not affect  **ERK**  activation.  Ref: 9763608 J Exp Med, 1998
- This study uses human alveolar macrophages to determine whether activation of a phosphatidylcholine PC specific phospholipase C PC  **PLC**  is linked to activation of the p42 44  **ERK**  kinases by LPS.  Ref: 10072552 J Immunol, 1999
- Similar to cooperation of Ras with PKC activation in  **ERK**  response, both  **PLC**  gamma2 dependent signal and GTPase are required for BCR induced JNK and p38 responses.  Ref: 9763608 J Exp Med, 1998
- It also activates or inhibits PKCs, PLD, PLA2, PC  **PLC** , nitric oxide synthase, and the  **ERK**  and SAPK JNK signaling cascades.  Ref: 10656539 Crit Rev Clin Lab Sci, 1999
- Reversion of v Ras or PC  **PLC**  induced transformation by expression of dominant negative lambdaPKC abolished the nuclear  **ERK**  activation suggesting lambdaPKC as a novel, direct or indirect, activator of mitogen activated protein kinase  **ERK**  kinase in response to activated Ras or elevated levels of phosphatidylcholine derived diacylglycerol.  Ref: 9111071 J Biol Chem, 1997
- These results indicate that P2Y receptors in astrocytes are coupled independently to PI  **PLC**  calcium and  **ERK**  pathways and suggest that signaling from P2Y receptors to  **ERK**  involves a calcium independent PKC isoform and hydrolysis of phosphatidylcholine by phospholipase D.  Ref: 10341225 J Neurosci, 1999
- Thus, RasGRP links TCR and  **PLC**  gamma1 to Ras  **Erk**  signaling, a pathway amenable to pharmacologic manipulation.  Ref: 10807788 Blood, 2000
- The study of molecules such as LAT, SLP 76, FYB, SKAP 55 and VAV have revealed multiple mechanisms with which to control the activation of downstream signaling pathways through RAS,  **PLC**  gamma 1 and  **ERK**  MAPK.  Ref: 10643561 Cell Calcium, 1999
- Intriguingly, overexpression of the SH3 domain of  **PLC**  gamma1, lipase inactive  **PLC**  gamma1, or wild type  **PLC**  gamma1 elevated p21Ras activity and  **ERK**  activity when compared with vector transfected cells.  Ref: 10913276 Biochemistry, 2000
- Moreover, PD98059 inhibited the expression of cytokines induced by LPS or PC  **PLC** , thus suggesting that  **ERK**  activity is also required for macrophage activation by these two agents.  Ref: 10702314 J Biol Chem, 2000
- As an aggregate, these studies support the hypothesis that one way by which LPS activates the  **ERK**  kinases is via activation of PC  **PLC** .  Ref: 10072552 J Immunol, 1999
- We show that TGF beta1 suppresses PDGF stimulated tyrosine phosphorylation of  **PLC**  gamma1, and the phosphorylation of  **Erk** .  Ref: 9252243 Life Sci, 1997
